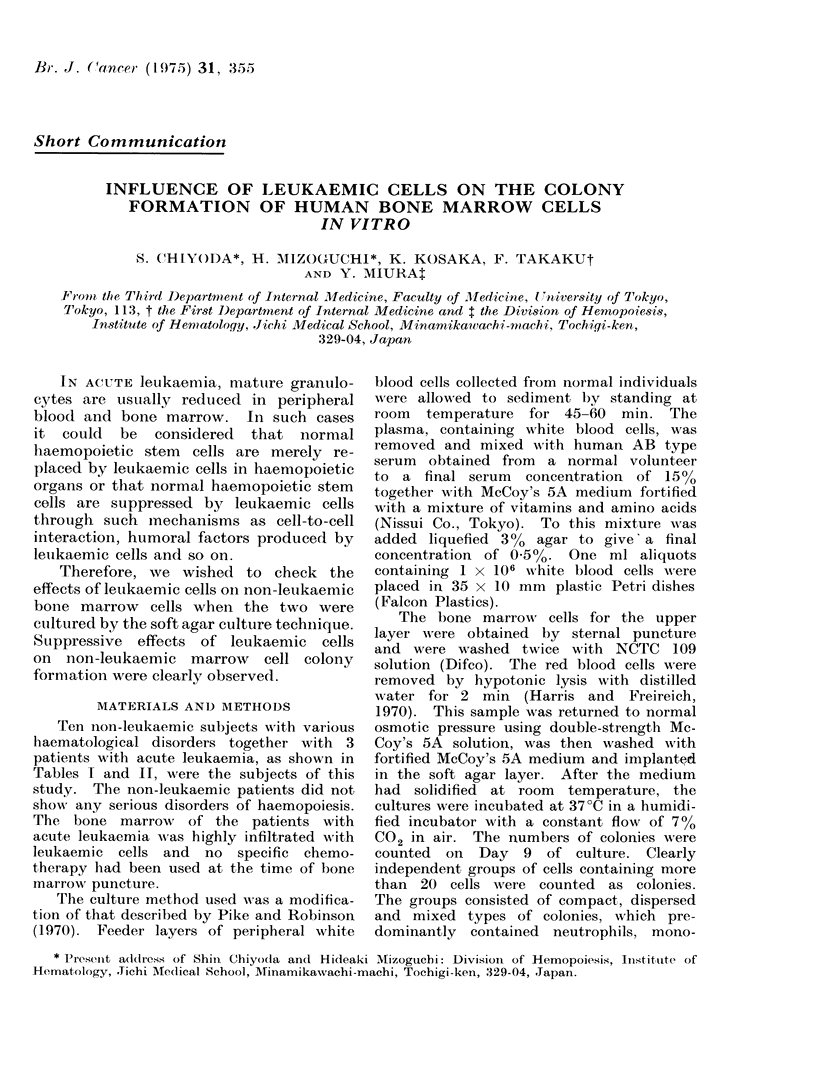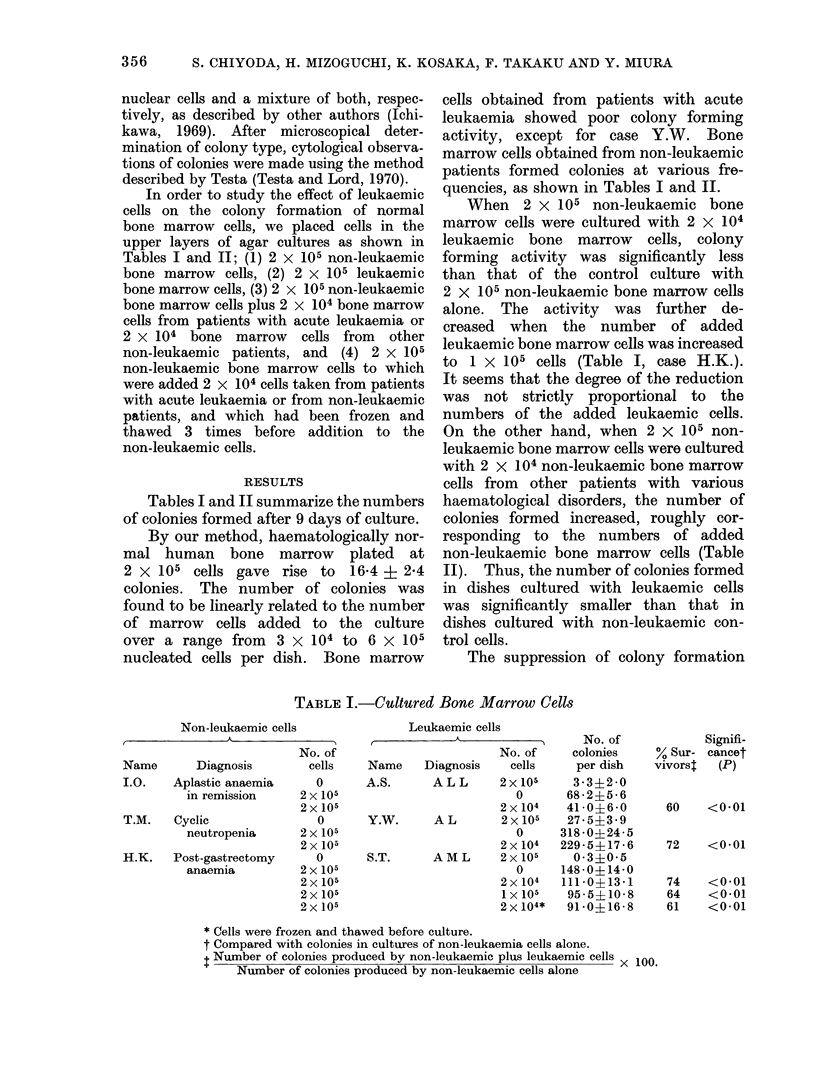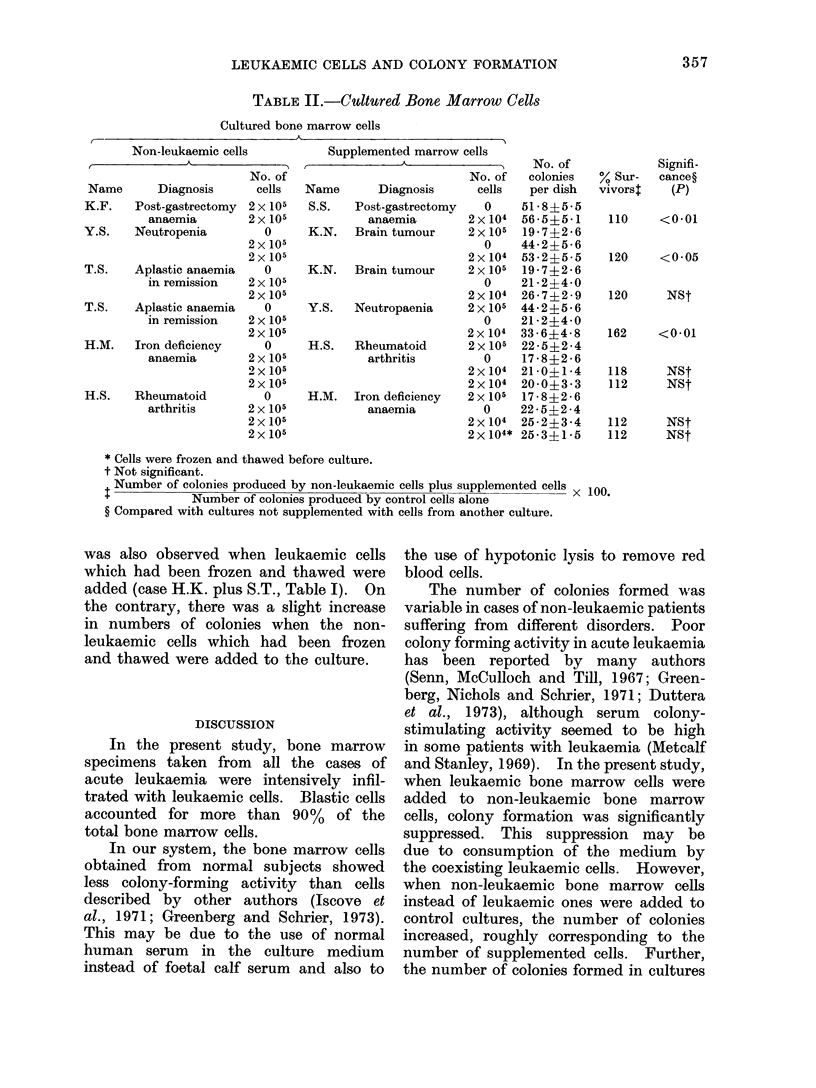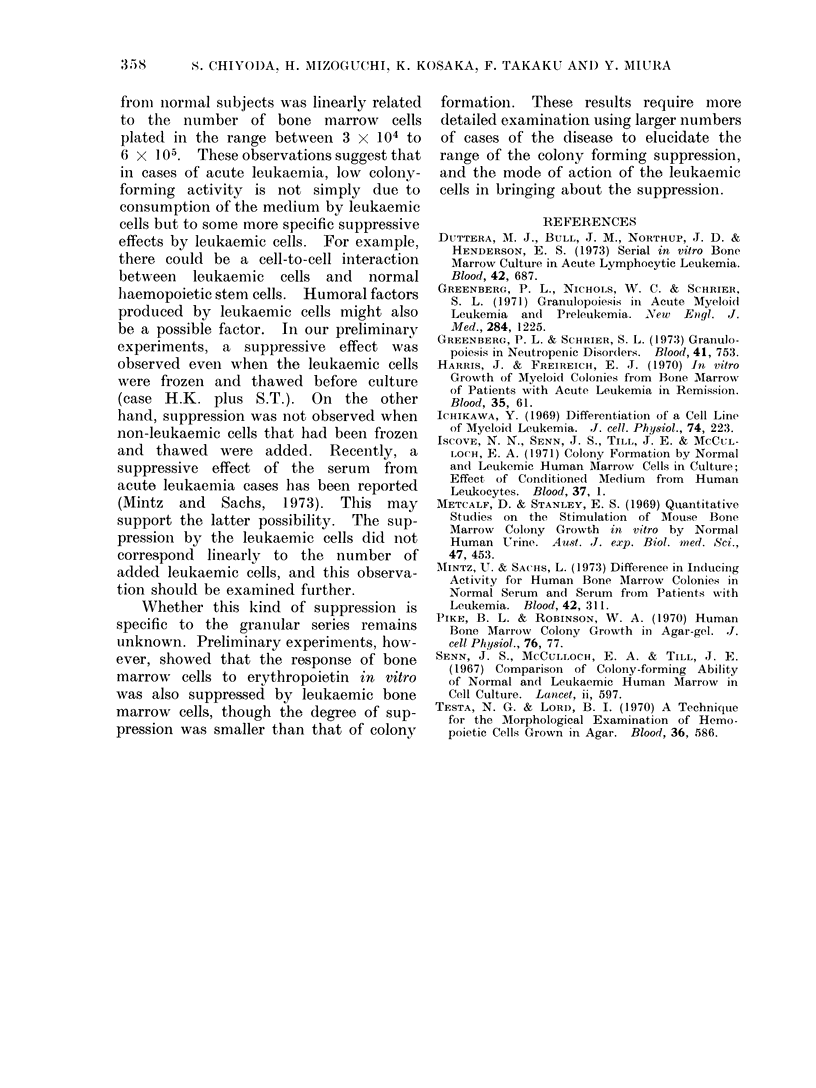# Influence of leukaemic cells on the colony formation of human bone marrow cells in vitro.

**DOI:** 10.1038/bjc.1975.70

**Published:** 1975-03

**Authors:** S. Chiyoda, H. Mizoguchi, K. Kosaka, F. Takaku, Y. Miura


Br. J. Cancer (1975) 31, 355

*Short Communication*

### INFLUENCE OF LEUKAEMIC CELLS ON THE COLONY FORMATION OF HUMAN BONE MARROW CELLS *IN VITRO*

S. CHIYODA*, H. MIZOGUCHI*, K. KOSAKA, F. TAKAKU† AND Y. MIURA‡

*From the Third Department of Internal Medicine, Faculty of Medicine, University of Tokyo, Tokyo, 113, † the First Department of Internal Medicine and ‡ the Division of Hemopoiesis, Institute of Hematology, Jichi Medical School, Minamikawachi-machi, Tochigi-ken, 329-04, Japan*

IN ACUTE leukaemia, mature granulocytes are usually reduced in peripheral blood and bone marrow. In such cases it could be considered that normal haemopoietic stem cells are merely replaced by leukaemic cells in haemopoietic organs or that normal haemopoietic stem cells are suppressed by leukaemic cells through such mechanisms as cell-to-cell interaction, humoral factors produced by leukaemic cells and so on.

Therefore, we wished to check the effects of leukaemic cells on non-leukaemic bone marrow cells when the two were cultured by the soft agar culture technique. Suppressive effects of leukaemic cells on non-leukaemic marrow cell colony formation were clearly observed.

### MATERIALS AND METHODS

Ten non-leukaemic subjects with various haematological disorders together with 3 patients with acute leukaemia, as shown in Tables I and II, were the subjects of this study. The non-leukaemic patients did not show any serious disorders of haemopoiesis. The bone marrow of the patients with acute leukaemia was highly infiltrated with leukaemic cells and no specific chemotherapy had been used at the time of bone marrow puncture.

The culture method used was a modification of that described by Pike and Robinson (1970). Feeder layers of peripheral white blood cells collected from normal individuals were allowed to sediment by standing at room temperature for 45–60 min. The plasma, containing white blood cells, was removed and mixed with human AB type serum obtained from a normal volunteer to a final serum concentration of 15% together with McCoy's 5A medium fortified with a mixture of vitamins and amino acids (Nissui Co., Tokyo). To this mixture was added liquefied 3% agar to give a final concentration of 0·5%. One ml aliquots containing $1 \times 10^6$ white blood cells were placed in 35 × 10 mm plastic Petri dishes (Falcon Plastics).

The bone marrow cells for the upper layer were obtained by sternal puncture and were washed twice with NCTC 109 solution (Difco). The red blood cells were removed by hypotonic lysis with distilled water for 2 min (Harris and Freireich, 1970). This sample was returned to normal osmotic pressure using double-strength McCoy's 5A solution, was then washed with fortified McCoy's 5A medium and implanted in the soft agar layer. After the medium had solidified at room temperature, the cultures were incubated at 37°C in a humidified incubator with a constant flow of 7% $CO_2$ in air. The numbers of colonies were counted on Day 9 of culture. Clearly independent groups of cells containing more than 20 cells were counted as colonies. The groups consisted of compact, dispersed and mixed types of colonies, which predominantly contained neutrophils, mono-

* Present address of Shin Chiyoda and Hideaki Mizoguchi: Division of Hemopoiesis, Institute of Hematology, Jichi Medical School, Minamikawachi-machi, Tochigi-ken, 329-04, Japan.

nuclear cells and a mixture of both, respectively, as described by other authors (Ichikawa, 1969). After microscopical determination of colony type, cytological observations of colonies were made using the method described by Testa (Testa and Lord, 1970).

In order to study the effect of leukaemic cells on the colony formation of normal bone marrow cells, we placed cells in the upper layers of agar cultures as shown in Tables I and II; (1) $2 \times 10^5$ non-leukaemic bone marrow cells, (2) $2 \times 10^5$ leukaemic bone marrow cells, (3) $2 \times 10^5$ non-leukaemic bone marrow cells plus $2 \times 10^4$ bone marrow cells from patients with acute leukaemia or $2 \times 10^4$ bone marrow cells from other non-leukaemic patients, and (4) $2 \times 10^5$ non-leukaemic bone marrow cells to which were added $2 \times 10^4$ cells taken from patients with acute leukaemia or from non-leukaemic patients, and which had been frozen and thawed 3 times before addition to the non-leukaemic cells.

### RESULTS

Tables I and II summarize the numbers of colonies formed after 9 days of culture.

By our method, haematologically normal human bone marrow plated at $2 \times 10^5$ cells gave rise to $16 \cdot 4 \pm 2 \cdot 4$ colonies. The number of colonies was found to be linearly related to the number of marrow cells added to the culture over a range from $3 \times 10^4$ to $6 \times 10^5$ nucleated cells per dish. Bone marrow cells obtained from patients with acute leukaemia showed poor colony forming activity, except for case Y.W. Bone marrow cells obtained from non-leukaemic patients formed colonies at various frequencies, as shown in Tables I and II.

When $2 \times 10^5$ non-leukaemic bone marrow cells were cultured with $2 \times 10^4$ leukaemic bone marrow cells, colony forming activity was significantly less than that of the control culture with $2 \times 10^5$ non-leukaemic bone marrow cells alone. The activity was further decreased when the number of added leukaemic bone marrow cells was increased to $1 \times 10^5$ cells (Table I, case H.K.). It seems that the degree of the reduction was not strictly proportional to the numbers of the added leukaemic cells. On the other hand, when $2 \times 10^5$ non-leukaemic bone marrow cells were cultured with $2 \times 10^4$ non-leukaemic bone marrow cells from other patients with various haematological disorders, the number of colonies formed increased, roughly corresponding to the numbers of added non-leukaemic bone marrow cells (Table II). Thus, the number of colonies formed in dishes cultured with leukaemic cells was significantly smaller than that in dishes cultured with non-leukaemic control cells.

The suppression of colony formation

TABLE I.—*Cultured Bone Marrow Cells*

| Non-leukaemic cells | | | Leukaemic cells | | | | | |
|---|---|---|---|---|---|---|---|---|
| Name | Diagnosis | No. of cells | Name | Diagnosis | No. of cells | No. of colonies per dish | % Survivors‡ | Significance† ($P$) |
| I.O. | Aplastic anaemia | 0 | A.S. | A L L | $2 \times 10^5$ | $3 \cdot 3 \pm 2 \cdot 0$ | | |
| | in remission | $2 \times 10^5$ | | | 0 | $68 \cdot 2 \pm 5 \cdot 6$ | | |
| | | $2 \times 10^5$ | | | $2 \times 10^4$ | $41 \cdot 0 \pm 6 \cdot 0$ | 60 | $<0 \cdot 01$ |
| T.M. | Cyclic | 0 | Y.W. | A L | $2 \times 10^5$ | $27 \cdot 5 \pm 3 \cdot 9$ | | |
| | neutropenia | $2 \times 10^5$ | | | 0 | $318 \cdot 0 \pm 24 \cdot 5$ | | |
| | | $2 \times 10^5$ | | | $2 \times 10^4$ | $229 \cdot 5 \pm 17 \cdot 6$ | 72 | $<0 \cdot 01$ |
| H.K. | Post-gastrectomy | 0 | S.T. | A M L | $2 \times 10^5$ | $0 \cdot 3 \pm 0 \cdot 5$ | | |
| | anaemia | $2 \times 10^5$ | | | 0 | $148 \cdot 0 \pm 14 \cdot 0$ | | |
| | | $2 \times 10^5$ | | | $2 \times 10^4$ | $111 \cdot 0 \pm 13 \cdot 1$ | 74 | $<0 \cdot 01$ |
| | | $2 \times 10^5$ | | | $1 \times 10^5$ | $95 \cdot 5 \pm 10 \cdot 8$ | 64 | $<0 \cdot 01$ |
| | | $2 \times 10^5$ | | | $2 \times 10^4$* | $91 \cdot 0 \pm 16 \cdot 8$ | 61 | $<0 \cdot 01$ |

* Cells were frozen and thawed before culture.
† Compared with colonies in cultures of non-leukaemia cells alone.
‡ $\frac{\text{Number of colonies produced by non-leukaemic plus leukaemic cells}}{\text{Number of colonies produced by non-leukaemic cells alone}} \times 100.$

TABLE II.—*Cultured Bone Marrow Cells*

| Cultured bone marrow cells: Non-leukaemic cells | | | Supplemented marrow cells | | | | | |
|---|---|---|---|---|---|---|---|---|
| Name | Diagnosis | No. of cells | Name | Diagnosis | No. of cells | No. of colonies per dish | % Survivors‡ | Significance§ ($P$) |
| K.F. | Post-gastrectomy | $2\times10^5$ | S.S. | Post-gastrectomy | 0 | $51\cdot8\pm5\cdot5$ | | |
| | anaemia | $2\times10^5$ | | anaemia | $2\times10^4$ | $56\cdot5\pm5\cdot1$ | 110 | $<0\cdot01$ |
| Y.S. | Neutropenia | 0 | K.N. | Brain tumour | $2\times10^5$ | $19\cdot7\pm2\cdot6$ | | |
| | | $2\times10^5$ | | | 0 | $44\cdot2\pm5\cdot6$ | | |
| | | $2\times10^5$ | | | $2\times10^4$ | $53\cdot2\pm5\cdot5$ | 120 | $<0\cdot05$ |
| T.S. | Aplastic anaemia | 0 | K.N. | Brain tumour | $2\times10^5$ | $19\cdot7\pm2\cdot6$ | | |
| | in remission | $2\times10^5$ | | | 0 | $21\cdot2\pm4\cdot0$ | | |
| | | $2\times10^5$ | | | $2\times10^4$ | $26\cdot7\pm2\cdot9$ | 120 | NS† |
| T.S. | Aplastic anaemia | 0 | Y.S. | Neutropaenia | $2\times10^5$ | $44\cdot2\pm5\cdot6$ | | |
| | in remission | $2\times10^5$ | | | 0 | $21\cdot2\pm4\cdot0$ | | |
| | | $2\times10^5$ | | | $2\times10^4$ | $33\cdot6\pm4\cdot8$ | 162 | $<0\cdot01$ |
| H.M. | Iron deficiency | 0 | H.S. | Rheumatoid | $2\times10^5$ | $22\cdot5\pm2\cdot4$ | | |
| | anaemia | $2\times10^5$ | | arthritis | 0 | $17\cdot8\pm2\cdot6$ | | |
| | | $2\times10^5$ | | | $2\times10^4$ | $21\cdot0\pm1\cdot4$ | 118 | NS† |
| | | $2\times10^5$ | | | $2\times10^4$ | $20\cdot0\pm3\cdot3$ | 112 | NS† |
| H.S. | Rheumatoid | 0 | H.M. | Iron deficiency | $2\times10^5$ | $17\cdot8\pm2\cdot6$ | | |
| | arthritis | $2\times10^5$ | | anaemia | 0 | $22\cdot5\pm2\cdot4$ | | |
| | | $2\times10^5$ | | | $2\times10^4$ | $25\cdot2\pm3\cdot4$ | 112 | NS† |
| | | $2\times10^5$ | | | $2\times10^4$* | $25\cdot3\pm1\cdot5$ | 112 | NS† |

* Cells were frozen and thawed before culture.
† Not significant.
‡ $\frac{\text{Number of colonies produced by non-leukaemic cells plus supplemented cells}}{\text{Number of colonies produced by control cells alone}} \times 100.$
§ Compared with cultures not supplemented with cells from another culture.

was also observed when leukaemic cells which had been frozen and thawed were added (case H.K. plus S.T., Table I). On the contrary, there was a slight increase in numbers of colonies when the non-leukaemic cells which had been frozen and thawed were added to the culture.

### DISCUSSION

In the present study, bone marrow specimens taken from all the cases of acute leukaemia were intensively infiltrated with leukaemic cells. Blastic cells accounted for more than 90% of the total bone marrow cells.

In our system, the bone marrow cells obtained from normal subjects showed less colony-forming activity than cells described by other authors (Iscove *et al.*, 1971; Greenberg and Schrier, 1973). This may be due to the use of normal human serum in the culture medium instead of foetal calf serum and also to the use of hypotonic lysis to remove red blood cells.

The number of colonies formed was variable in cases of non-leukaemic patients suffering from different disorders. Poor colony forming activity in acute leukaemia has been reported by many authors (Senn, McCulloch and Till, 1967; Greenberg, Nichols and Schrier, 1971; Duttera *et al.*, 1973), although serum colony-stimulating activity seemed to be high in some patients with leukaemia (Metcalf and Stanley, 1969). In the present study, when leukaemic bone marrow cells were added to non-leukaemic bone marrow cells, colony formation was significantly suppressed. This suppression may be due to consumption of the medium by the coexisting leukaemic cells. However, when non-leukaemic bone marrow cells instead of leukaemic ones were added to control cultures, the number of colonies increased, roughly corresponding to the number of supplemented cells. Further, the number of colonies formed in cultures from normal subjects was linearly related to the number of bone marrow cells plated in the range between $3 \times 10^4$ to $6 \times 10^5$. These observations suggest that in cases of acute leukaemia, low colony-forming activity is not simply due to consumption of the medium by leukaemic cells but to some more specific suppressive effects by leukaemic cells. For example, there could be a cell-to-cell interaction between leukaemic cells and normal haemopoietic stem cells. Humoral factors produced by leukaemic cells might also be a possible factor. In our preliminary experiments, a suppressive effect was observed even when the leukaemic cells were frozen and thawed before culture (case H.K. plus S.T.). On the other hand, suppression was not observed when non-leukaemic cells that had been frozen and thawed were added. Recently, a suppressive effect of the serum from acute leukaemia cases has been reported (Mintz and Sachs, 1973). This may support the latter possibility. The suppression by the leukaemic cells did not correspond linearly to the number of added leukaemic cells, and this observation should be examined further.

Whether this kind of suppression is specific to the granular series remains unknown. Preliminary experiments, however, showed that the response of bone marrow cells to erythropoietin *in vitro* was also suppressed by leukaemic bone marrow cells, though the degree of suppression was smaller than that of colony formation. These results require more detailed examination using larger numbers of cases of the disease to elucidate the range of the colony forming suppression, and the mode of action of the leukaemic cells in bringing about the suppression.

### REFERENCES

DUTTERA, M. J., BULL, J. M., NORTHUP, J. D. & HENDERSON, E. S. (1973) Serial *in vitro* Bone Marrow Culture in Acute Lymphocytic Leukemia. *Blood*, **42**, 687.

GREENBERG, P. L., NICHOLS, W. C. & SCHRIER, S. L. (1971) Granulopoiesis in Acute Myeloid Leukemia and Preleukemia. *New Engl. J. Med.*, **284**, 1225.

GREENBERG, P. L. & SCHRIER, S. L. (1973) Granulopoiesis in Neutropenic Disorders. *Blood*, **41**, 753.

HARRIS, J. & FREIREICH, E. J. (1970) *In vitro* Growth of Myeloid Colonies from Bone Marrow of Patients with Acute Leukemia in Remission. *Blood*, **35**, 61.

ICHIKAWA, Y. (1969) Differentiation of a Cell Line of Myeloid Leukemia. *J. cell. Physiol.*, **74**, 223.

ISCOVE, N. N., SENN, J. S., TILL, J. E. & MCCULLOCH, E. A. (1971) Colony Formation by Normal and Leukemic Human Marrow Cells in Culture; Effect of Conditioned Medium from Human Leukocytes. *Blood*, **37**, 1.

METCALF, D. & STANLEY, E. S. (1969) Quantitative Studies on the Stimulation of Mouse Bone Marrow Colony Growth *in vitro* by Normal Human Urine. *Aust. J. exp. Biol. med. Sci.*, **47**, 453.

MINTZ, U. & SACHS, L. (1973) Difference in Inducing Activity for Human Bone Marrow Colonies in Normal Serum and Serum from Patients with Leukemia. *Blood*, **42**, 311.

PIKE, B. L. & ROBINSON, W. A. (1970) Human Bone Marrow Colony Growth in Agar-gel. *J. cell Physiol.*, **76**, 77.

SENN, J. S., MCCULLOCH, E. A. & TILL, J. E. (1967) Comparison of Colony-forming Ability of Normal and Leukaemic Human Marrow in Cell Culture. *Lancet*, ii, 597.

TESTA, N. G. & LORD, B. I. (1970) A Technique for the Morphological Examination of Hemopoietic Cells Grown in Agar. *Blood*, **36**, 586.